# Evaluation of the CARBA PAcE test, a colorimetric imipenem hydrolysis test for rapid detection of carbapenemase activity

**DOI:** 10.1128/spectrum.00891-24

**Published:** 2024-10-23

**Authors:** Nadya Rakovitsky, Mor N. Lurie-Weinberger, Elizabeth Temkin, Amichay Hameir, Reut Efrati-Epchtien, Liat Wulffhart, Alona Keren Paz, David Schwartz, Yehuda Carmeli

**Affiliations:** 1 National Institute for Antibiotic Resistance and Infection Control, Israel Ministry of Health, Tel Aviv, Israel; 2 School of Medicine, Tel Aviv University, Tel Aviv, Israel; Ross University School of Veterinary Medicine, Basseterre, Saint Kitts and Nevis, Caribbean

**Keywords:** carbapenem resistance, carbapenemase, imipenem hydrolysis test, colorimetric test, CARBA PAcE, β CARBA

## Abstract

There is an urgent need for accurate and fast diagnostic tests to identify carbapenemase-producing bacteria. Here, we evaluated a colorimetric imipenem hydrolysis test, called the CARBA PAcE test, to detect carbapenemase-producing Gram-negative bacteria (GNB). We tested a collection of 270 GNB isolates with a characterized carbapenemase content. Our testing set included 205 carbapenemase-producing, carbapenemase-resistant *Enterobacterales* (*CP CRE*) with 40 *Klebsiella pneumoniae* carbapenemase (KPC), 49 New Delhi metallo beta lactamase (NDM), 49 OXA-48-like, 15 Verona integron-mediated metallo-β-lactamase (VIM), three IMP, 43 IMI, six isolates producing more than one carbapenemase, and 65 non-carbapenemase-producing *Enterobacterales* (20 ESBL producers, 35 non-carbapenemase-producing, carbapenem-resistant [non-CP CRE], and 10 carbapenem-susceptible *Enterobacterales* [non-CP, non-CSE, third-generation cephalosporin and carbapenem susceptible]). We compared the performances of the CARBA PAcE test, the qualitative colorimetric β-CARBA test, and the modified CarbaNP test to a gold standard of carbapenemase gene detection by PCR. Specificities of all tests were high: 95.4% (62/65) for CARBA PAcE test, 98.5% (64/65) for β-CARBA test, and 100% (65/65) for the modified CarbaNP test. Sensitivity varied by carbapenemase: all three tests had a sensitivity of 100% for NDM, VIM, and IMP and 97.5% for KPC. Sensitivity to detect IMI was 0% for the CARBA PAcE and β-CARBA tests and 11.6% for the modified CarbaNP test. Sensitivity to detect OXA-48-like was 89.7% for the CARBA PAcE test, 87.7% for the β-CARBA test, and 14.2% for the modified CarbaNP test. Reading the results of the CARBA PAcE assay was difficult. The CARBA PAcE assay is highly sensitive for detecting NDM, VIM, IMP, and KPC, but slightly less sensitive for OXA-48-like. It does not detect IMI. It is highly specific, and its overall diagnostic accuracy is similar to that of β-CARBA. Its operational advantages are rapid turnaround time, ease of use, and long shelf life, but reading of results is subjective.

**IMPORTANCE:**

We evaluated the ability of the CARBA PAcE test to detect carbapenemases in 274 Gram-negative isolates with a known carbapenemase content. Specificity was high for all carbapenemases tested (96.9%). Sensitivity was high for KPC, NDM, VIM, and IMP (97.5-100%); but lower for OXA-48-like (89.7%). Activity of IMI could not be detected. Taken together, our results indicate that CARBA PAcE is a useful alternative in regions where NDM and KPC are predominant. The limitations of the test are difficulty in reading results and incompatibility with mSuperCARBA.

## INTRODUCTION

Rapid detection of carbapenemase-producing Gram-negative bacteria (CP-GNB) is important for guiding appropriate antibiotic treatment and timely and effective infection control measures. Gene detection, mostly using PCR, is the gold standard for diagnosis. However, the utility of PCR may be diminished by carbapenemase mutations (1, 2), rendering them undetectable by PCR (3). Similarly, PCR may miss isolates carrying less common carbapenemases that are not included in the testing panel. Moreover, a PCR can produce a false positive result if a mutation affects the carbapenemase activity, but not the primer binding sites/gene length. Therefore, some guidelines advise using a phenotypic test to supplement PCR. There are several biochemical methods that can be used to detect the carbapenemase activity, including qualitative colorimetric testing assays (4, 5), such as β-CARBA (Bio-Rad, Marne la Coquette, France), RAPIDEC CARBA-NP (bioMérieux, France), carbapenem inactivation method (CIM) (6), and imipenem hydrolysis (7). To be useful globally, a clinical test must be able to detect most common carbapenemases, including NDM, KPC, VIM, IMP, IMI, and OXA-48-like. Colorimetric assays are easy to use and commonly employed by clinical microbiology laboratories. However, these tests have significant limitations. RAPIDEC CARBA-NP produces false negative results in mucoid strains and for enzymes with a low carbapenemase activity, such as OXA-48-like (5). β-CARBA has a very short shelf life and, like RAPIDEC CARBA-NP, has a low sensitivity for OXA-48-like variants (4). Here, we evaluated the performance of a new colorimetric imipenem hydrolysis kit, called CARBPA PAcE, and compared it to the performances of the β-CARBA test and the modified CarbaNP test.

## MATERIALS AND METHODS

### Sample

Clinical GNB isolates were selected from the collection of the National Institute for Antibiotic Resistance, Israel to represent the normal distribution of the clinical samples from 2017 to 2023 in Israel. Identification of isolates and antimicrobial susceptibility testing were performed using VITEK 2 (bioMérieux, France). Genomic DNA was extracted using a Genomic DNA Purification Kit (Promega, WI, USA) following the manufacturer’s instructions. The mechanism of carbapenem resistance was determined by multiplex PCR for *bla*_KPC_, *bla*_OXA-48-like_, *bla*_NDM_, *bla*_IMP_, *bla*_VIM_, and *bla*_IMI_ (2) based on the Israeli National Policy for CPE Screening (8–11). The multiplex PCR was performed in a total volume of 20 µL with the following conditions: initial denaturation at 95°C for 5 min; 30 cycles of 94°C for 30 s; 60°C for 60 s; 72°C for 60 s; and a final extension at 72°C for 4 min. The PCR results served as the gold standard to which the CARBA PAcE assay (MAST Group, Bootle, UK), the β-CARBA assay (Bio-Rad, Marne la Coquette, France) (4), and the modified CarbaNP test were compared (12, 13). Bacterial isolates were grown on blood agar, Muller Hinton agar (MHA), and CHROMagar mSuperCARBA (HyLab, Rehovot, Israel). All isolates were stored at −80°C, sub-cultured on blood agar plates at 37°C, and transferred twice prior to testing.

### CARBA PAcE

CARBA PAcE tests were performed according to the manufacturer’s instructions. Briefly, bacterial isolates were cultured overnight at 37°C on blood agar. A full inoculation loop (approximately 5 µL) of bacteria was transferred to 250 uL of lysis buffer and vortexed for 20 s until full homogenization. Turbidity was adjusted to 3 McFarland standard. After incubation at 37°C for 10 min, color changes were visually evaluated according to the manufacturer’s instructions. In the case of the discrepancy between PCR and CARBA PAcE results, the CARBA PAcE test was repeated twice, and the consistent result was used. Positive (ATCC1705) and negative (ATCC1706) controls were used in each experiment.

### β-CARBA

β-CARBA tests were performed according to the manufacturer’s instructions. Briefly, bacterial isolates were cultured overnight at 37°C on blood agar. A full inoculation loop (approximately 5 µL) of bacteria was transferred to the mix and vortexed for 20 s until full homogenization. Results were read after incubation at 37°C for 30 min. ATCC1705 was used as a positive control and ATCC1706 as a negative control in each experiment. The color was re-evaluated after 90 min. The test was repeated if the results were not consistent with previous readings.

### Modified CarbaNP

Modified CarbaNP imipenem hydrolysis assay based on CARBA NP assay (13, 14) with a number of adjustments (incubation volume, incubation time, ZnSO_4_ concentration). Bacterial isolates were cultured overnight on blood agar. A full inoculation loop (approximately 5 µL) of bacteria was re-suspended in 200 µL of bacterial protein extraction reagent (B-PER, Thermo Fisher Scientific, USA) and vortexed for 1 min until full homogenization. After incubation at room temperature for 30 min, the solution was centrifuged for 5 min at 10,000 rpm. A mixture of phenol red (Sigma Aldrich, Rehovot, Israel) and 5 µM ZnS0_4_xH_2_O (Sigma Aldrich, Rehovot, Israel) was prepared with and without imipenem/cilastatin (6 mg/mL, 50% active compound). The sample (30 µL) was incubated with 100 µL of phenol red mixture and stored in a dark box for a maximum of 2 h at 37°C, followed by visual examination. In each experiment, ATCC1705 was used as a positive control and ATCC1706 as a negative control.

### Meropenem minimum inhibitory concentration (MIC)

The meropenem MIC of all isolates (Table S1) was determined by ETEST (bioMérieux, France) according to the manufacturer’s instructions. In brief, the overnight inoculum was adjusted to 0.5 McFarland and spread on an MH plate, and an ETEST strip was placed on the surface. After incubation overnight at 37°C, the meropenem MIC was interpreted in accordance with the European Committee on Antimicrobial Susceptibility Testing (EUCAST) guidelines v 13.1 (15).

### Statistical analysis

Sensitivity, specificity, and their 95% confidence intervals (CI) were calculated using PCR results as the gold standard. Isolates harboring more than one type of carbapenemase were included in the calculation of overall sensitivity but not in the subgroup analysis by carbapenemase. Confidence intervals were calculated in VassarStats (http://vassarstats.net/prop1.html).

### Simulation analysis

The prevalence of OXA-48-like and non-CP CRE in the general population affects the percentage of false results for each test. Therefore, we calculated false negative and false positive rates under scenarios of 1,000 isolates that differed by (i) the proportion of CRE that is CPE and (ii) the proportion of CPE that is OXA-48-like. False negatives refer to OXA-48-like isolates that were not identified as OXA-48-like. False positives refer to non-CPE isolates that were incorrectly identified as CPE. We focused on OXA-48-like because the test sensitivity for the other common carbapenemases was above 97%. We examined different combinations of CPE prevalence (60–90%) and OXA-48-like prevalence among CPE (5%–50%). We performed these calculations for the CARBA-PAcE test using the sensitivity and specificity that were determined in this study.

## RESULTS

**Fig 1:**
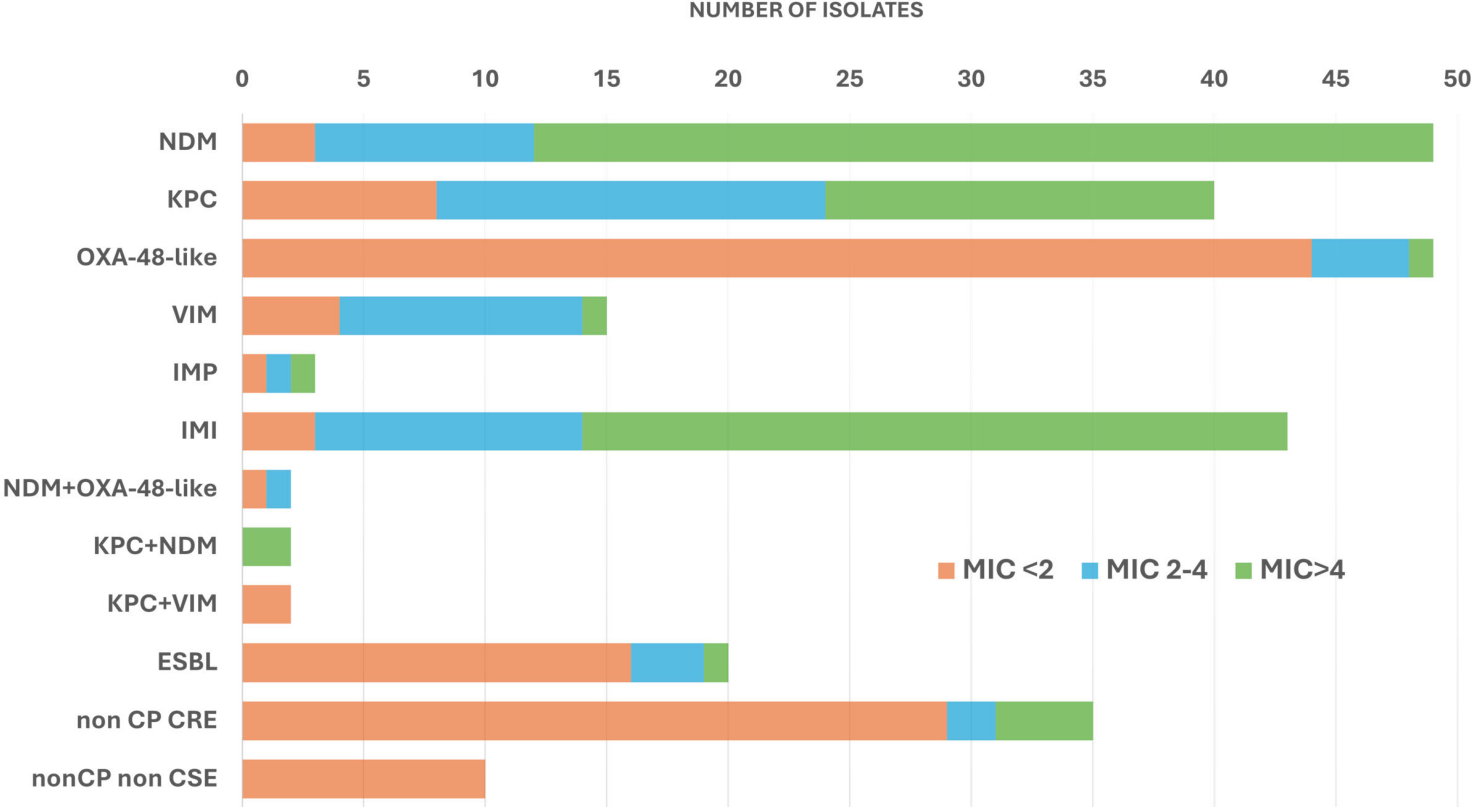
Meropenem MIC (mg/L) ranges of isolates according to their carbapenemase type and status. The meropenem MIC is determined by ETEST (bioMérieux, France).

The sample consisted of 270 GNB isolates: 205 carbapenemase-producing *Enterobacterales* (CP CRE), 20 extended-spectrum beta-lactamase (ESBL) producers (18 CTX-M-15, one CTX-M-39, and one CTX-M-14), 35 non-carbapenemase-producing, carbapenem-resistant *Enterobacterales* (non-CP CRE), and 10 carbapenem-susceptible *Enterobacterales* (CSE, third-generation cephalosporin and carbapenem susceptible). The distribution of species and carbapenemases is shown in Table 1. Meropenem MIC (mg/L) distributions for each carbapenemase mechanism are shown in Fig. 1.

**TABLE 1:** Description of isolates used in this study

		CP CRE									
Organism tested	No. of isolates	KPC	NDM	OXA-48-like	VIM	IMP	IMI	Combinations	ESBL	Non-CP CRE^*c*^	Non-CP non-CSE
*Klebsiella pneumoniae*	84	23	13		1				19	26	2
*Escherichia coli*	95	11	21	48				NDM + OXA-48- like (1)		8	6
*Enterobacter cloacae*	68	2	6		14		41	KPC + NDM (1)	1	1	2
*Klebsiella oxytoca*	2	1						NDM + OXA-48- like (1)			
*Citrobacter amalonaticus*	2	1	1								
*Citrobacter freundii*	6	2	3			1					
*Enterobacter homachei*	1		1								
*Raoultella planticola*	2		2								
*Pseudocitrobacter faecalis*	1							KPC + NDM (1)			
*Enterobacter* spp.	1						1				
*Enterobacter ludwigii*	1						1				
*Kluyvera cryocrescens*	2							KPC + VIM (2)			
*Klebsiella aerogenes*	1			1							
*Proteus mirables*	1		1								
*Serratia marcescens*	3		1			2					
Total	270	40	49	49	15	3	43	6	20	35	10

^*a*^ Non-carbapenemase producing (non-CP) refers to common carbapenemases.

^*b*^ Non-CP CRE: non-carbapenemase-producing carbapenem-resistant *Enterobacterales.*

^*c*^ Common carbapenemases not detected.

**Fig 2:**
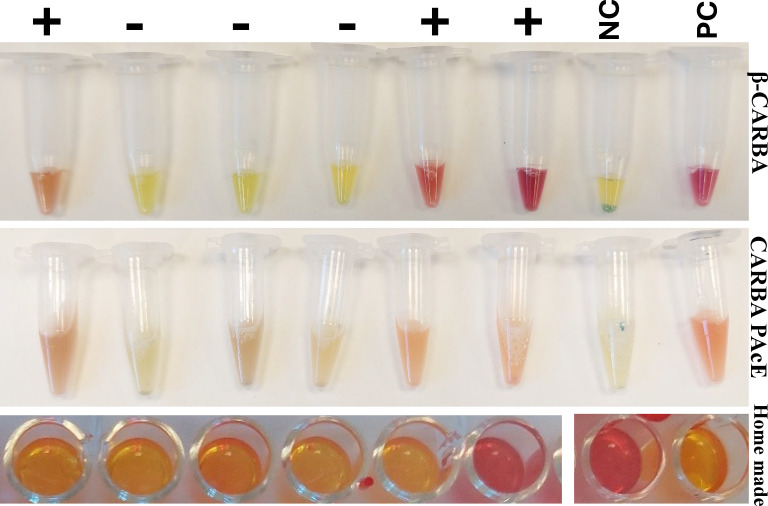
Color range in the β-CARBA, Carba-PAcE, and modified CarbaNP tests. Negative control ATCC1706, positive control ATCC1705. Positive (+) and negative (−) samples according to the presence of the checked carbapenemases by PCR. Shown samples are different *Escherichia coli* OXA-48-like isolates. NC = negative control; PC = positive control.

Three hydrolysis tests were compared, with all isolates sub-cultured on blood agar as per manufacturers’ instructions. The interpretation of the results was the easiest for β-CARBA, as the colors were stronger and more distinguishable (Fig. 2). A detailed comparison of the sensitivities of the three tests by carbapenemase type is shown in Table 2A.

**TABLE 2:** Comparison of CARBA-PAcE, β-CARBA, and modified CarbaNP tests for detection of carbapenemase production by carbapenemase type: (A) sensitivity and (B) specificity

	Sensitivity % [95% CI]					
Carbapenemase type	CARBA-PAcE		β-CARBA		Modified CarbaNP	
	%	95% CI	%	95% CI	%	95% CI
Class A (*n* = 83)	46.9% (39/83)	[35.9–58.3]	46.9% (39/83)	[35.9–58.3]	53.0% (44/83)	[41.7–64.1]
IMI (*n* = 43)	0% (0/43)	[0.0–8.2]	0% (0/43)	[0.0–8.2]	11.6% (5/43)	[5.0–24.4]
KPC (*n* = 40)	97.5% (39/40)	[87.1–99.5]	97.5% (39/40)	[87.1–99.5]	97.5% (39/40)	[87.1–99.5]
Class B (*n* = 67)	100% (67/67)	[94.5–100.0]	100% (67/67)	[94.5–100.0]	100% (67/67)	[94.5–100.0]
NDM (*n* = 49)	100% (49/49)	[92.7–100.0]	100% (49/49)	[92.7–100.0]	100% (49/49)	[92.7–100.0]
VIM (*n* = 15)	100% (15/15)	[79.6–100.0]	100% (15/15)	[79.6–100.0]	100% (15/15)	[79.6–100.0]
IMP (*n* = 3)	100% (3/3)	[43.8–100.0]	100% (3/3)	[43.8–100.0]	100% (3/3)	[43.8–100.0]
Class D (*n* = 49)	89.7% (44/49)	[78.2–95.5]	87.7% (43/49)	[75.7–94.2]	14.2% (7/49)	[7.1–26.6]
OXA-48-like (*n* = 49)	89.7% (44/49)	[78.2–95.5]	87.7% (43/49)	[75.7–94.2]	14.2% (7/49)	[7.1–26.6]
Combinations (*n* = 6)	100% (6/6)	[60.1–100.0]	100% (6/6)	[60.1–100.0]	83.3 (5/6)	[43.6–96.9]
Summary	75.6% (155/205)	[69.1–81.3]	76.1% (156/205)	[69.7–81.8]	60.0% (123/205)	[52.9–66.7]

	Specificity % [95% CI]					
Carbapenemase type	CARBA-PAcE		β-CARBA		Home-made	
	%	95% CI	%	95% CI	%	95% CI
ESBL	100% (20/20)	[83.9–100]	100% (20/20)	[83.9–100]	100% (20/20)	[83.9–100]
Non-CP CRE^*a*^	91.4% (32/35)	[76.9–98.2]	97.1% (34/35)	[ 85.0–99.9]	100% (35/35)	[90.0–100.0]
Non-CP non-CSE	100% (10/10)	[72.2–100]	100% (10/10)	[72.2–100]	100% (10/10)	[72.2–100]
Summary	95.4% (62/65)	[87.1–99.0]	98.5% (64/65)	[91.7–99.9]	100.0% (65/65)	[94.5–100.0]

^*a*^ Common carbapenemases not detected.

The sensitivity of all methods was the highest (100%) for Class B β-lactamases (NDM, VIM, IMP) and for KPC. False-negative results occurred for OXA-48-like in all three tests. Both β-CARBA and CARBA PAcE performed adequately in OXA-48-like producer detection, correctly identifying 87.8% (43/49) and 89.8% (44/49) isolates, respectively. In contrast, the modified CarbaNP test detected only 14.2% (7/49) OXA-48-like producers.

CARBA PAcE and β-CARBA did not detect any of the IMI producers (0% sensitivity), while the modified CarbaNP test detected 1s1.6% (5/43).

Specificity was 95.4% (62/65) for CARBA PAcE, 100% (64/65) for β-CARBA, and 100% (65/65) (Table 2B) for the modified CarbaNP test. Isolates considered as true negatives by the gold standard PCR were 20 ESBL isolates (18 CTX-M-15, one CTX-M-39, and one CTXM-14), 10 CSE, and 35 non-CP CRE isolates (Table 1). Apparently, false-positive results were noted for three non-CP CRE: two *E. coli* and one *K. pneumoniae*.

**Fig 3:**
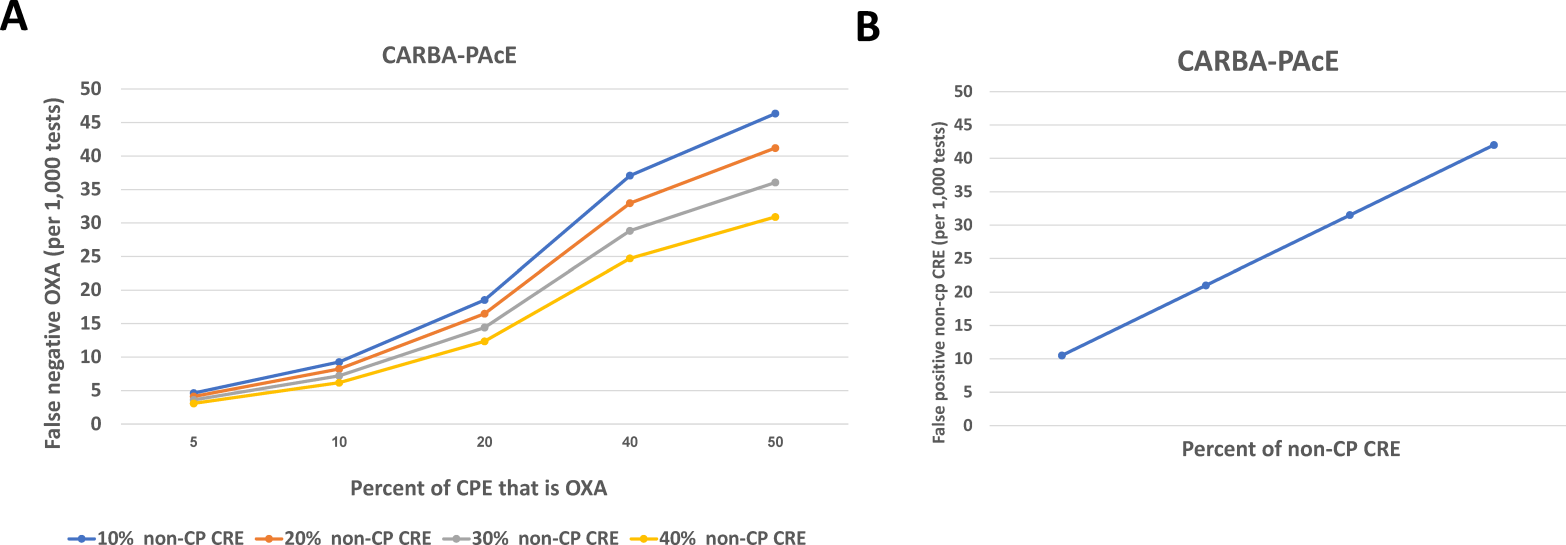
Association between local epidemiology of CRE and CARBA-PAcE test performance. (**A**) Effect of the prevalence of CPE among CRE and OXA-48-like among carbapenemases on the false-negative rate. (**B**) Effect of the prevalence of non-CP CRE among CRE on the false-positive rate.

Fig. 3A and B show how the false-negative and false-positive rates vary depending on the prevalence of CPE among CRE and the prevalence of OXA-48-like among CPE. As these rise, the false negative rate rises (Fig. 3A). For example, when 90% of CRE is CPE, and 50% of CPE is OXA-48-like, the false negative rate is 46%. Likewise, as the prevalence of non-CP CRE among CRE rises, the false positive rate rises (Fig. 3B).

## DISCUSSION

CARBA PAcE has a number of advantages over β-CARBA and modified CarbaNP tests (Table 3). It has the longest shelf life of 4 months compared to 1 month for β-CARBA. In contrast, the modified CarbaNP test has to be freshly prepared before each use. The CARBA PAcE test requires less hands-on time when compared with the β-CARBA and modified CarbaNP tests, thereby providing the shortest TAT and reducing potential errors.

**TABLE 3:** Comparison of the practical aspects of the three tests for detecting carbapenemases

Method	Test shelf life	Number of steps	Time to result	Cost/isolate (relative^*a*^)	Expertise required
Modified CarbaNP test	24 h	3	Up to 2 h	X	Intermediate
β-CARBA	1 month	2	30 min	6X	Low
CARBA PAcE	4 month	1	10 min	3X	Low

^*a*^ Price as offered at 2023 in Israel.

The overall performances of the β-CARBA and CARBA PAcE tests were similar: for both tests, the sensitivity was 97.5% for KPC and 100% for NDM. The sensitivity of CARBA PAcE in our study was comparable to that reported in previous studies: 72.0% (16) and 79.8% (17). Both commercial kits outperformed the modified CarbaNP test, which had a sensitivity of 60.0%. All tests were specific, with the specificity of 96.9% for CARBA PAcE, 100% for β-CARBA, and 100% for the modified CarbaNP test.

The sensitivity reported here was lower than that reported for RAPIDEC CARBA NP (bioMerieux, France) (98%) and for Neo-Rapid CARB (Rosco) (89%) (18). However, that study included only two IMI isolates with 100% reported sensitivity. In our study, the lower sensitivity was driven primarily by 43 IMI isolates, and none of which were detected by β-CARBA or CARBA PAcE. The poor sensitivity for IMI detection probably stems from the lower hydrolyzing activity of IMI enzymes (19).

Among the findings, one KPC isolate with a MIC = 2 mg/L was a false negative in all three tests, and one NDM isolate with a MIC = 0.25 mg/L was a false negative in the modified CarbaNP test. Additionally, five OXA-48-like isolates tested as false negative with the CARBA PAcE Kit, all having MICs lower than 0.5 mg/L, and six OXA-48-like samples tested false negative with the β-CARBA kit, all with MICs lower than 1 mg/L. However, due to the limited sample size, the statistical significance of the correlation between false negative results with MIC reading cannot be established.

The sensitivity for all three tests for rare metallo β-lactamases, such as VIM and IMP, was 100%; however, the sample size is not statistically significant (15 VIM isolates and three IMP isolates). The CARBA-PAcE and β-CARBA tests failed to detect rare β-lactamase IMI (0% sensitivity), while the modified CarbaNP test identified it in 11.6% (five out of 43) cases probably due to the known weak hydrolyzing activity of this carbapenemase type (20). However, Mancini et al. (20) demonstrated that the RAPIDEC CARBA NP test (bioMerieux, France) achieved 100% sensitivity in detecting IMI.

False positive results were noted in 8.5% (3/35) of carbapenem-resistant, non-carbapenemase-producing isolates. False positive results due to ESBL production have been observed in other colorimetric tests (4). Possibly, these false positive results could reflect the presence of an unidentified, rare carbapenemase.

*Serratia marcescens* is acknowledged as a species of concern in the CARBA PAcE manual. *S. marcescens* can produce colored pigments that may hamper the specificity of the test. We tested three *S. marcescens* isolates (those isolates were not pigmented), one producing NDM and two producing IMP, and all three were accurately detected. We did not test non-carbapenemase-producing or ESBL-producing *S. marcescens*; therefore, we cannot confirm the problematic production of the pigments that may reduce CARBA PAcE specificity.

Among the disadvantages of CARBA PAcE, we found that the colors indicating the results can be difficult to distinguish (Fig. 2), a problem also reported by Rezzoug et al. (17). In addition, CARBA PAcE cannot be used on isolates cultured on CHROMagar mSuperCARBA, as CHROMagar mSuperCARBA is a colorimetric medium, leading to interference with the reading of assay results. The incompatibility of CARBA PAcE with CHROMagar mSuperCARBA media is a significant limitation since many clinical microbiology laboratories use CHROMagar mSuperCARBA for CPE detection. Sub-culturing on blood agar would delay the results by at least 16 h.

This study has several limitations. The sample does not represent the epidemiological distribution of carbapenemases in Israel. Rare carbapenemases were intentionally over-represented. Therefore, performance in routine diagnostic tests, where the most common carbapenemases (NDM, KPC, and OXA-48-like) prevail, is expected to be better than reported here. Another limitation was the medium selection, which affected the result of the assay. We used blood agar for its compatibility with CARBA PAcE, but it does not reflect routine laboratory practices, where mSuperCARBA media may be streamlined with workflow. Finally, all CARBA PAcE results, in which the color was uncertain, were classified as positive, which is subjective.

In conclusion, while CARBA PAcE test showed overall acceptable performance for the detection of carbapenemases in the studied isolates, with the sensitivity of 75.6% and the specificity of 95.4%, it was highly sensitive and specific for the most common carbapenemases. When compared to the β-CARBA test, CARBA PAcE provided similar results, with slightly higher sensitivity, but lower specificity. For laboratories with a high prevalence of NDM, KPC, and OXA-48-like carbapenemases, CARBA PAcE may provide a valuable and time-saving alternative. When the local epidemiological situation shows a predominance of CPE (compared to non-CPE), and most of the CPE are OXA-48-like carbapenemases, the use of CARBA PAcE is less advisable due to the high likelihood of false negative and false positive results. The test’s advantages include long shelf life, ease of use, and rapid turnaround time of only 10 min. However, the colors indicating results can be difficult to distinguish, and CARBA PAcE cannot be used with isolates cultured on CHROMagar mSuperCARBA, posing a limitation for routine diagnostic use. Therefore, it is essential to consider the compatibility with the isolates' culture medium and be cautious when interpreting ambiguous color results.

## SUPPLEMENTAL MATERIAL

Table 1 spectrum.00891-24-s0001.xlsx Strain used in this study.

ASM does not own the copyrights to Supplemental Material that may be linked to, or accessed through, an article. The authors have granted ASM a non-exclusive, world-wide license to publish the Supplemental Material files. Please contact the corresponding author directly for reuse.
